# Supplementary material for: Pyruvate Dehydrogenase Kinase 1 inhibition mediated oxidative phosphorylation enhancement in cartilage promotes osteoarthritis progression
Source: BMC Musculoskelet Disord. 2023 Jul 20;24:597. doi: 10.1186/s12891-023-06585-6 (PMC10357736; doi:10.1186/s12891-023-06585-6)
Supplement: Supplementary file 1 — Additional file 1. [file 12891_2023_6585_MOESM1_ESM.zip › KEGG.Gsea.1653622432464/KEGG_GLYCOLYSIS_GLUCONEOGENESIS.html]

Details for gene set KEGG\_GLYCOLYSIS\_GLUCONEOGENESIS[GSEA]

|  || Dataset | 55457\_collapsed\_to\_symbols.cls.cls #OA\_versus\_normal.cls.cls #OA\_versus\_normal\_repos |
| Phenotype | cls.cls#OA\_versus\_normal\_repos |
| Upregulated in class | OA |
| GeneSet | KEGG\_GLYCOLYSIS\_GLUCONEOGENESIS |
| Enrichment Score (ES) | 0.34036967 |
| Normalized Enrichment Score (NES) | 1.3979143 |
| Nominal p-value | 0.03943662 |
| FDR q-value | 0.11402852 |
| FWER p-Value | 0.957 |
Table: GSEA Results Summary

  

Fig 1: Enrichment plot: KEGG\_GLYCOLYSIS\_GLUCONEOGENESIS      
 Profile of the Running ES Score & Positions of GeneSet Members on the Rank Ordered List

  

| SYMBOL | TITLE | RANK IN GENE LIST | RANK METRIC SCORE | RUNNING ES | CORE ENRICHMENT || 1 | PDHB | pyruvate dehydrogenase E1 subunit beta [Source:HGNC Symbol;Acc:HGNC:8808] | 149 | 0.862 | 0.0470 | Yes |
| 2 | GPI | glucose-6-phosphate isomerase [Source:HGNC Symbol;Acc:HGNC:4458] | 166 | 0.842 | 0.1030 | Yes |
| 3 | PGAM1 | phosphoglycerate mutase 1 [Source:HGNC Symbol;Acc:HGNC:8888] | 341 | 0.727 | 0.1388 | Yes |
| 4 | DLD | dihydrolipoamide dehydrogenase [Source:HGNC Symbol;Acc:HGNC:2898] | 565 | 0.635 | 0.1646 | Yes |
| 5 | PCK2 | "phosphoenolpyruvate carboxykinase 2, mitochondrial [Source:HGNC Symbol;Acc:HGNC:8725]" | 759 | 0.579 | 0.1889 | Yes |
| 6 | FBP1 | fructose-bisphosphatase 1 [Source:HGNC Symbol;Acc:HGNC:3606] | 772 | 0.576 | 0.2271 | Yes |
| 7 | AKR1A1 | aldo-keto reductase family 1 member A1 [Source:HGNC Symbol;Acc:HGNC:380] | 808 | 0.567 | 0.2629 | Yes |
| 8 | ALDOC | "aldolase, fructose-bisphosphate C [Source:HGNC Symbol;Acc:HGNC:418]" | 870 | 0.551 | 0.2956 | Yes |
| 9 | ALDH2 | aldehyde dehydrogenase 2 family member [Source:HGNC Symbol;Acc:HGNC:404] | 1003 | 0.517 | 0.3204 | Yes |
| 10 | ALDH3A2 | aldehyde dehydrogenase 3 family member A2 [Source:HGNC Symbol;Acc:HGNC:403] | 1263 | 0.465 | 0.3319 | Yes |
| 11 | ALDH7A1 | aldehyde dehydrogenase 7 family member A1 [Source:HGNC Symbol;Acc:HGNC:877] | 2158 | 0.343 | 0.2856 | Yes |
| 12 | PFKM | "phosphofructokinase, muscle [Source:HGNC Symbol;Acc:HGNC:8877]" | 2260 | 0.331 | 0.3002 | Yes |
| 13 | BPGM | bisphosphoglycerate mutase [Source:HGNC Symbol;Acc:HGNC:1093] | 2398 | 0.315 | 0.3110 | Yes |
| 14 | PDHA1 | pyruvate dehydrogenase E1 subunit alpha 1 [Source:HGNC Symbol;Acc:HGNC:8806] | 2433 | 0.310 | 0.3294 | Yes |
| 15 | PGK2 | phosphoglycerate kinase 2 [Source:HGNC Symbol;Acc:HGNC:8898] | 2566 | 0.295 | 0.3392 | Yes |
| 16 | ALDH9A1 | aldehyde dehydrogenase 9 family member A1 [Source:HGNC Symbol;Acc:HGNC:412] | 2795 | 0.270 | 0.3398 | Yes |
| 17 | PGAM2 | phosphoglycerate mutase 2 [Source:HGNC Symbol;Acc:HGNC:8889] | 3008 | 0.249 | 0.3402 | Yes |
| 18 | ENO3 | enolase 3 [Source:HGNC Symbol;Acc:HGNC:3354] | 3207 | 0.230 | 0.3404 | Yes |
| 19 | FBP2 | fructose-bisphosphatase 2 [Source:HGNC Symbol;Acc:HGNC:3607] | 3763 | 0.180 | 0.3094 | No |
| 20 | HK3 | hexokinase 3 [Source:HGNC Symbol;Acc:HGNC:4925] | 3792 | 0.178 | 0.3193 | No |
| 21 | PKM | pyruvate kinase M1/2 [Source:HGNC Symbol;Acc:HGNC:9021] | 3963 | 0.159 | 0.3169 | No |
| 22 | PGM1 | phosphoglucomutase 1 [Source:HGNC Symbol;Acc:HGNC:8905] | 3990 | 0.157 | 0.3255 | No |
| 23 | TPI1 | triosephosphate isomerase 1 [Source:HGNC Symbol;Acc:HGNC:12009] | 4059 | 0.150 | 0.3304 | No |
| 24 | ALDOA | "aldolase, fructose-bisphosphate A [Source:HGNC Symbol;Acc:HGNC:414]" | 4289 | 0.129 | 0.3213 | No |
| 25 | ADH5 | "alcohol dehydrogenase 5 (class III), chi polypeptide [Source:HGNC Symbol;Acc:HGNC:253]" | 4720 | 0.094 | 0.2943 | No |
| 26 | ADH1C | "alcohol dehydrogenase 1C (class I), gamma polypeptide [Source:HGNC Symbol;Acc:HGNC:251]" | 4908 | 0.079 | 0.2851 | No |
| 27 | LDHAL6B | lactate dehydrogenase A like 6B [Source:HGNC Symbol;Acc:HGNC:21481] | 5040 | 0.070 | 0.2796 | No |
| 28 | G6PC1 | glucose-6-phosphatase catalytic subunit 1 [Source:HGNC Symbol;Acc:HGNC:4056] | 5389 | 0.041 | 0.2553 | No |
| 29 | PFKP | "phosphofructokinase, platelet [Source:HGNC Symbol;Acc:HGNC:8878]" | 5500 | 0.032 | 0.2490 | No |
| 30 | ENO2 | enolase 2 [Source:HGNC Symbol;Acc:HGNC:3353] | 5572 | 0.026 | 0.2452 | No |
| 31 | ALDH3B2 | aldehyde dehydrogenase 3 family member B2 [Source:HGNC Symbol;Acc:HGNC:411] | 5617 | 0.022 | 0.2433 | No |
| 32 | PFKL | "phosphofructokinase, liver type [Source:HGNC Symbol;Acc:HGNC:8876]" | 5632 | 0.021 | 0.2437 | No |
| 33 | DLAT | dihydrolipoamide S-acetyltransferase [Source:HGNC Symbol;Acc:HGNC:2896] | 5647 | 0.020 | 0.2440 | No |
| 34 | ALDH1A3 | aldehyde dehydrogenase 1 family member A3 [Source:HGNC Symbol;Acc:HGNC:409] | 5831 | 0.007 | 0.2302 | No |
| 35 | ALDH3B1 | aldehyde dehydrogenase 3 family member B1 [Source:HGNC Symbol;Acc:HGNC:410] | 5862 | 0.004 | 0.2281 | No |
| 36 | ALDH3A1 | aldehyde dehydrogenase 3 family member A1 [Source:HGNC Symbol;Acc:HGNC:405] | 6413 | -0.036 | 0.1878 | No |
| 37 | ALDH1B1 | aldehyde dehydrogenase 1 family member B1 [Source:HGNC Symbol;Acc:HGNC:407] | 6551 | -0.046 | 0.1802 | No |
| 38 | HK1 | hexokinase 1 [Source:HGNC Symbol;Acc:HGNC:4922] | 6728 | -0.058 | 0.1704 | No |
| 39 | LDHC | lactate dehydrogenase C [Source:HGNC Symbol;Acc:HGNC:6544] | 7603 | -0.127 | 0.1110 | No |
| 40 | LDHB | lactate dehydrogenase B [Source:HGNC Symbol;Acc:HGNC:6541] | 7718 | -0.135 | 0.1114 | No |
| 41 | ALDOB | "aldolase, fructose-bisphosphate B [Source:HGNC Symbol;Acc:HGNC:417]" | 8421 | -0.187 | 0.0694 | No |
| 42 | PGK1 | phosphoglycerate kinase 1 [Source:HGNC Symbol;Acc:HGNC:8896] | 8513 | -0.195 | 0.0755 | No |
| 43 | ADH7 | "alcohol dehydrogenase 7 (class IV), mu or sigma polypeptide [Source:HGNC Symbol;Acc:HGNC:256]" | 8906 | -0.225 | 0.0603 | No |
| 44 | ADH1B | "alcohol dehydrogenase 1B (class I), beta polypeptide [Source:HGNC Symbol;Acc:HGNC:250]" | 9036 | -0.235 | 0.0662 | No |
| 45 | G6PC2 | glucose-6-phosphatase catalytic subunit 2 [Source:HGNC Symbol;Acc:HGNC:28906] | 9088 | -0.239 | 0.0785 | No |
| 46 | PCK1 | phosphoenolpyruvate carboxykinase 1 [Source:HGNC Symbol;Acc:HGNC:8724] | 9100 | -0.239 | 0.0939 | No |
| 47 | PDHA2 | pyruvate dehydrogenase E1 subunit alpha 2 [Source:HGNC Symbol;Acc:HGNC:8807] | 9124 | -0.241 | 0.1085 | No |
| 48 | ADH1A | "alcohol dehydrogenase 1A (class I), alpha polypeptide [Source:HGNC Symbol;Acc:HGNC:249]" | 9283 | -0.254 | 0.1135 | No |
| 49 | ADH6 | alcohol dehydrogenase 6 (class V) [Source:HGNC Symbol;Acc:HGNC:255] | 9504 | -0.274 | 0.1150 | No |
| 50 | ENO1 | enolase 1 [Source:HGNC Symbol;Acc:HGNC:3350] | 9606 | -0.284 | 0.1264 | No |
| 51 | HK2 | hexokinase 2 [Source:HGNC Symbol;Acc:HGNC:4923] | 10030 | -0.325 | 0.1156 | No |
| 52 | PKLR | pyruvate kinase L/R [Source:HGNC Symbol;Acc:HGNC:9020] | 10120 | -0.333 | 0.1313 | No |
| 53 | GCK | glucokinase [Source:HGNC Symbol;Acc:HGNC:4195] | 10137 | -0.335 | 0.1528 | No |
| 54 | LDHA | lactate dehydrogenase A [Source:HGNC Symbol;Acc:HGNC:6535] | 11134 | -0.448 | 0.1057 | No |
| 55 | GAPDH | glyceraldehyde-3-phosphate dehydrogenase [Source:HGNC Symbol;Acc:HGNC:4141] | 11268 | -0.467 | 0.1270 | No |
Table: GSEA details [plain text format]

  

Fig 2: KEGG\_GLYCOLYSIS\_GLUCONEOGENESIS      
 Blue-Pink O' Gram in the Space of the Analyzed GeneSet

  

Fig 3: KEGG\_GLYCOLYSIS\_GLUCONEOGENESIS: Random ES distribution      
 Gene set null distribution of ES for **KEGG\_GLYCOLYSIS\_GLUCONEOGENESIS**
